# A Longitudinal Assessment of Vocabulary Retention in Symbol-Competent Chimpanzees (*Pan troglodytes*)

**DOI:** 10.1371/journal.pone.0118408

**Published:** 2015-02-23

**Authors:** Michael J. Beran, Lisa A. Heimbauer

**Affiliations:** 1 Language Research Center, Georgia State University, University Plaza, Atlanta, Georgia, United States of America; 2 Department of Psychology, The Pennsylvania State University, Moore Building, University Park, State College, Pennsylvania, United States of America; University of Portsmouth, UNITED KINGDOM

## Abstract

A number of studies from the 1960s to 1990s assessed the symbolic competence of great apes and other animals. These studies provided varying forms of evidence that some species were capable of symbolically representing their worlds, both through productive symbol use and comprehension of symbolic stimuli. One such project at the Language Research Center involved training chimpanzees (*Pan troglodytes*) to use lexigram symbols (geometric visual stimuli that represented objects, actions, locations, and individuals). Those studies now are more than 40 years old, and only a few of the apes involved in those studies are still alive. Three of these chimpanzees (and a fourth, control chimpanzee) were assessed across a 10-year period from 1999 to 2008 for their continued knowledge of lexigram symbols and, in the case of one chimpanzee, the continued ability to comprehend human speech. This article describes that longitudinal assessment and outlines the degree to which symbol competence was retained by these chimpanzees across that decade-long period. All chimpanzees showed retention of lexigram vocabularies, although there were differences in the number of words that were retained across the individuals. One chimpanzee also showed continual retention of human speech perception. These retained vocabularies largely consisted of food item names, but also names of inedible objects, locations, individuals, and some actions. Many of these retained words were for things that are not common in the daily lives of the chimpanzees and for things that are rarely requested by the chimpanzees. Thus, the early experiences of these chimpanzees in symbol-rich environments have produced long-lasting memories for symbol meaning, and those competencies have benefited research in a variety of topics in comparative cognition.

## Introduction

Ape language research has its early roots in cross-fostering studies conducted in the early twentieth century [1–2]. In these early studies, chimpanzees (*Pan troglodytes*) were raised by human caregivers with the research focus on speech acquisition, production, and symbol use. The results of these studies demonstrated that despite cross-fostering efforts the chimpanzees were unable to exhibit any speech production capabilities. It is now known that this inability is partly due to morphological constraints [3], although there is evidence that some animals, including birds, elephants, and harbor seals, demonstrate speech imitation abilities [4–7]. Additionally, in some cases, the speech production by birds may show some of the referential aspects that are related to language [5, 8].

Later studies made use of chimpanzees’ and other apes’ natural gestural capabilities to investigate their aptitude to communicate using American Sign Language [9–12], tokens [13] and other referential symbols such as lexigrams [14–18]. Sign language studies with the chimpanzee Washoe, and later with several other chimpanzees, revealed that these animals used sign language to communicate much like children, including two-way interactions and evidence for use of sentence constituents [19–21]. Several vocabulary tests assessing correct sign usage, both gesturally and conceptually, revealed that Washoe, Tatu, Dar, and Moja, correctly responded to approximately 80%, 82%, 81%, and 54% of object photos, respectively [22]. These tests did not, however, assess retention. Testing was conducted around the time that these chimpanzees were approximately four years old and while their ASL acquisition and usage was still developing.

During the time these sign language studies were being conducted, a different kind of study emerged looking at the ability of a chimpanzee named Lana to learn and use a symbol system of geometric patterns called lexigrams. This study and those that followed were conducted at the Yerkes National Primate Research Center at Emory University [16], and then later at the Language Research Center (LRC) at Georgia State University. The Language Analogue (LANA) project sought to investigate Lana’s symbolic competence and the usefulness of a computer-controlled symbol-training system for individuals with limited language abilities [23]. Lana’s training began when she was a little over the age of two years in a highly structured environment with emphasis on her symbol production using a computerized keyboard. Her symbol usage focused on sentence-like structured requests and interactions. Although Lana did not demonstrate conceptual knowledge of the lexigrams [24], she did master the rule-based requirements of her computer communication system and learned to discern grammatically correct sequences from ungrammatical sequences. Even at the age of 27 years, she still retained knowledge of symbolic referents for approximately 20 objects [25].

Beginning in the 1970s at the Yerkes Primate Center, and then continuing at the LRC, two young male chimpanzees named Sherman and Austin also were trained with the lexigram system. Their training, however, was conducted in a more interactive environment than Lana’s with both humans and with each other. Sherman and Austin revealed greater symbol acquisition than Lana [18], and at the age of 25 Sherman retained the ability to identify approximately 35 items using lexigrams and photographs [26]. Sherman not only used lexigrams to identify objects but also to communicate his intentions, to ask for items that were absent from his immediate environment, and to demonstrate symbol-based, cross-modal matching with haptic and olfactory stimuli [27–29]. Although Sherman’s communication abilities demonstrated greater conceptual knowledge in regard to the items referenced by the lexigrams than Lana’s did, he did not demonstrate spoken English comprehension [18].

A subsequent LRC project investigated symbol learning and lexigram usage beginning in 1985 with a female chimpanzee named Panpanzee (Panzee). Panzee was co-reared with a female bonobo (*Pan paniscus*) named Panbanisha, and this project employed a different learning regimen. These two chimpanzees were raised from birth in a manner similar to humans, hearing and responding to speech from the age of eight days old [30–31]. The methodological approach was now one of learning about lexigrams within the context of symbol usage rather than symbol training, per se. For example, Panzee and Panbanisha were exposed to the meaning of these symbols, and corresponding speech, during daily routines, which included interactions with others and traveling within their indoor and outdoor environments. The result of this study was that these animals communicated using lexigrams as Lana, Sherman, and Austin did, but they also demonstrated strong English comprehension. Panzee, in particular, at the age of 12 was able to identify over 100 lexigrams or photographs. More impressively, she also was able to choose these visual referents when hearing the corresponding English word [26].

An influential factor in the difference among Lana’s, Sherman’s, and Panzee’s symbol usage and competence, and Panzee’s English comprehension abilities, is most likely due to their different rearing histories [17, 26, 32], which has also been cited as an important factor for symbol acquisition by bonobos [33–34]. As is the case with humans, immersion in a language-rich environment is a key component of symbol acquisition and successful communication skills. The human experience includes observational learning in language-specific situations in order to facilitate and solidify communication abilities, including spoken language comprehension.

There were also other important differences in rearing between Lana, Sherman, and Panzee—their age at the start of their respective projects. Lana was close to 2 years of age and came from the colony of chimpanzees at the Yerkes Primate Center to her new environment in which she was the only chimpanzee, and in which she interacted mainly with her computer system and a small number of humans. Sherman was mother-raised for 1.5 years, and then he lived in the chimpanzee nursery at the Yerkes Primate Center for 1 year before beginning the language project (which, at the time was still at the Yerkes Center). Sherman participated in the language project with another chimpanzee, Austin, with whom he lived for many years as part of that project. Panzee came to the Language Research Center within the first two weeks of her life, and as noted, she spent extensive periods of time with humans, and also with the bonobo Panbanisha. Thus, there were clear differences in training philosophy (including the methods and goals of each of the projects), but there were also differences in the ages at which the chimpanzees began in the projects, and the early experiences of these chimpanzees in terms of their time with their mothers and how extensively they interacted with humans and other apes. It remains unclear as to what extent each of these factors contributed to the resulting symbol abilities of these chimpanzees, although we believe all of these factors are critical, and other research has shown clearly and convincingly that the early rearing experiences of chimpanzees can have major positive (or negative) effects on their social and cognitive abilities [35].

Lana, Sherman, and Panzee continued to participate in cognitive research in the years after their formal language-acquisition projects ended (Lana and Sherman are still alive, and Panzee died in 2014). The Discussion section outlines briefly some of that recent and ongoing cognitive research. They all participated in the current longitudinal study to assess their retention of learned vocabulary over a period of 10 years. These animals interacted daily with one another and with their human caretakers and researchers by using lexigrams. Keyboards were mounted within their indoor enclosures and outdoor play yards. All three chimpanzees used their lexigram keyboards on a regular basis, albeit somewhat differently [26]. Lana used the communication system primarily to request food and drink items and in response to food preference questions. Sherman used the lexigrams to request food and answer food-related queries, but also to request to be moved to another area or to participate in activities (i.e., to play a game of “chase” or to watch a video). In addition to using the lexigram communication system as Sherman did, Panzee also sometimes commented about items and events in her surroundings.

As noted, Beran et al. [26] provided a single vocabulary retention test for the chimpanzees to compare their proficiency with lexigrams then to what was demonstrated when they were more formally engaged in ape language research. That report was, in essence, the starting point for this decade-long longitudinal assessment of the vocabulary retention of these three animals, as well as a fourth animal, Mercury, who has lived with the other three chimpanzees his entire life, but without any experience in symbol-training. Mercury is thus the control subject, although it is important to also remember that the rearing histories of Lana, Sherman, and Panzee were quite different from one another, and that too impacts their vocabulary retention. What we report here is the outcome of that decade-long assessment of vocabulary retention. The implications of ongoing symbolic competencies of these animals are also discussed, in addition to the relevance of symbol competence for broader assessments of chimpanzee learning and cognition. The continued symbolic competence of these chimpanzees affords unique experimental opportunities, and those opportunities rely on the present data to outline and define what symbols might be used in other cognitive task contexts.

## Methods

### Participants

The four chimpanzees, Lana (female, born 1971), Sherman (male, born 1973), Panzee (female, born 1985), and Mercury (male, born 1986) have been housed together since 1992. The rearing histories and symbolic competencies of these individuals have already been described above and elsewhere [16–18, 30–31]. The housing area in which these chimpanzees live consists of three indoor cages each approximately 3 m by 5 m and a fourth indoor cage approximately 6 m by 7 m. These cages were interconnected, and they also were connected to three outdoor yards, one of which was approximately 4 m by 6 m and two of which were each approximately 8 m by 15 m and that each had a 7 m high climbing tower. The group was socially housed in various combinations throughout the course of the experiment. Individuals were separated during a work session into one of the indoor cages. All of the chimpanzees were maintained on a regular diet of fruit, vegetables, and supplemental primate chow (Lab Fiber-Plus Monkey Diet, PMI Nutrition Intl, LLC, Brentwood, MO) throughout the test period, including test days. Water was available continuously. The chimpanzees worked on this task at their own choosing, and could end a test session whenever they wanted by leaving the test area.

### Materials

The task was presented on a personal computer (PC) running Windows operating system with attached monitor and joystick. The program used was a matching-to-sample program described below. Joysticks were attached to the indoor cages via ports connected to the front of each cage. This allowed the chimpanzees to place their hands into the ports to manipulate the joysticks. The computers and monitors were placed on rolling carts approximately 1 m in height. Monitors were at a distance of approximately 1 m from each participant during testing. Details on the general procedure and apparatus (the LRC-CTS) that is used for testing nonhuman primates at the LRC has been more thoroughly described elsewhere [36]. All four chimpanzees were highly proficient in using this system, as they performed a large number of computerized experiments before and during the course of this long-term study [37–42].

Chimpanzees were tested in this experiment once per year, with testing typically requiring between 2 and 6 weeks to complete, depending on what other experiments were concurrently conducted with the chimpanzees. This testing typically took place between the late fall and winter, and chimpanzees were tested from 3 to 5 times per week.

### General Procedure

At the beginning of each trial a circular cursor was centered in the bottom 1/3 of the monitor screen. In the center of the screen was the sample stimulus. Depending on the condition, this sample was either: 1) a photograph, 2) a lexigram, or 3) a spoken English word (that was played by the computer speaker when the chimpanzees touched a solid grey rectangle on the screen). Photographs were digital images of real world items, individuals, locations, or, in some cases, actions that typically were represented as two or more human actors in an interaction such as chasing each other. Lexigrams were the geometric icons that make up the lexigram keyboard used at the LRC and each represented a different item, action, location, or individual. The English samples were presented in the first author’s voice recorded as a .wav file. The list of test words used in these three formats throughout this experiment is shown in Table 1.

**Table 1:** The list of words used for testing the vocabularies of the chimpanzees separated into categories.

*Actions*					
Chase	Dig	Draw	Drink*	Fight	Grab
Groom	Hide	Hug	Hurt	Tickle	Wash
*Apes*					
Austin	Gorilla	Kanzi	Lana	Matata	Orangutan
Panbanisha	Panzee	Sherman	Tamuli		
*Body Parts*					
Foot	Hand	Head	Tummy		
*Foods*					
Apple	Apricot	Banana	Blackberries	Blueberries	Bread
Burrito	Butter	Candy*	Carrot	Celery	Cereal
Cheese	Cherries	Chicken	Chow	Clover	Coconut
Coffee	Coke	Egg	Grapes	Green beans	Hambur
Hotdog	Ice	Jello	Jelly	Juice	Honeysuckle
Kiwi	Koolaid	Leaf	Lemon	Lemonade	Lettuce
M&Ms	Melon	Milk	Noodles	Onion	Marshmallow*
Orange	Orange drink	Orange juice	Peaches	Peanut	Pear
Peas	Perrier	Pineapple	Popsicle	Potato	Pomegranate
Privet	Raisins	Salt	Sour cream	Strawberries	Sugar
Sugarcane	Surprise	Sweet potato	Taco	Tea*	Tomato
Vitamins	Water	Watermelon*	Yogurt		
*Locations*					
A-frame	Bedroom	Camper	Child side	Colony room	Crisscross
Flat rock	Group room	Gullygusher	Hilltop	Log cabin	Lookout
Middle test	Midway	MushTrail	NASA	Observation	Play yard
River	Sand pile	Scrubby pine	Shop	Staff office	Sue’s gate
Sue’s office	Tool room	Trailer	Tree house	T-room	
*Objects*					
Backpack	Ball	Balloon	Bark	Blanket	Book
Bowl	Box*	Brush*	Bubbles	Bug	Bunny
Cabinet	Camera	Can opener	Car	Chalk*	Clay
Clippers*	Collar	Cooler*	Crayon*	Dog	Fire
Hammer	Hat	Hose	Joystick*	Keyboard	Keys
Knife	Lever	Light	Lighter	Magnet	Medicine*
Mirror	Money	Nails	Nest	Oil	Paint
Paper	Phone	Pillow	Pinecone	Pine needle	Pinky
Plastic bag	Rain	Refrigerator	Rock	Rubber bank	Shirt
Shoe	Shot	Snake	Soap	Sparkler	Sponge
Spoon	Stethoscope	Stick	Straw	String	Thermometer
Toothbrush	Toothpaste	Towel	Trash	Turtle	Tv
Umbrella	Vacuum	Wipes			

Note. Words with asterisks (*) next to them were not part of the tests given in 1999–2003. They were included in 2004–2008 testing.

At the onset of each trial, the chimpanzees used the joystick to move the cursor into contact with the sample. If the sample was a photograph or lexigram, four match choices appeared in any of six different locations around the perimeter of the screen (three locations on each side of the screen). If the sample was an English word, the .wav file for that word was played when the chimpanzee touched a rectangle in the center of the screen with the cursor, and then the visual match choices (lexigrams or photographs) were presented in four of the six locations on the screen. A sample photograph or lexigram remained present throughout the trial.

The chimpanzee had to move the cursor into contact with a match choice to make a response. Correct selections were followed with a melodic tone and the presentation of the next trial. Incorrect selections were followed by a buzz tone and the presentation of the next trial (there was no time penalty for incorrect responses). The experimenter working with the chimpanzees gave a food reward after the majority of correct responses, but not after every correct response. Sometimes, two chimpanzees worked at the same time, in separate testing areas without visual access to each other’s computer screens, and the experimenter moved between them giving a food reward after one or more correct responses, and so the rewards were not given on a fixed schedule but were more somewhat intermittent. This led to the chimpanzees working at a fairly rapid pace, producing multiple trials per minute.

Critically, the experimenter could not see any of the computer screens during testing. Thus, he could not see the choice options available to the chimpanzees, nor their initial responses to any of those stimuli. He was completely blind to all aspects of the trial, except that on trials with spoken English samples he could hear the sample. However, he could not see the choice options, and thus could not cue the chimpanzees in any way during their responses. He only knew the outcome of the trial after the program provided auditory feedback.

It is important to note that this was the general procedure. However, across a decade of testing, some aspects varied. For example, in some years, only one investigator was available to test the chimpanzees during test sessions, and other years two experimenters might have each worked with a different chimpanzee at the same time but in different test locations within the building (and with the same controls for cuing in place that were described above). In addition, things such as the cage in which each chimpanzee worked often varied, as these animals changed over time in terms of their preferred working locations (there are four places where joysticks can be attached to the enclosures for testing). The animals’ diets also varied over the years, and so the food rewards used changed from year to year in terms of what was still approved by the veterinarian as a test reward. Of course, over a decade of testing, the computer equipment became damaged or obsolete and had to be replaced, usually with a more modern operating system, or a new model of joystick. However, the procedure outlined above was kept as stable as possible given these other necessary changes to the general procedure. It is important to note that the chimpanzees continued to work on the current computerized task as well as many other tasks despite the change in testing setup or equipment over the years.

### Experimental Conditions

**Fig 1:**
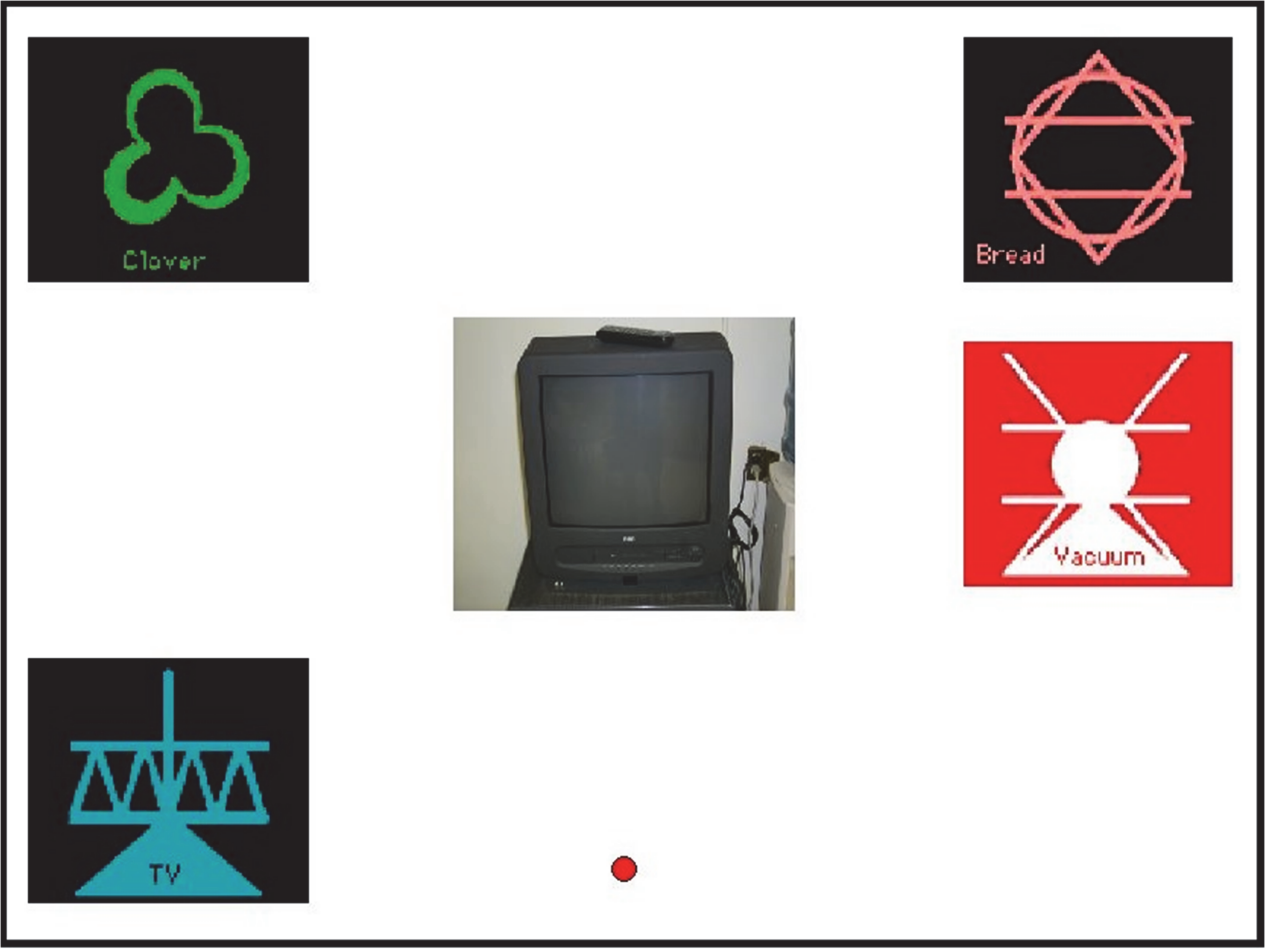
An example trial. The sample is at center (TV) and the four match choices are presented around the perimeter of the screen. The chimpanzee must move the cursor into contact with one of the match choices to make a selection. For Panzee, auditory samples were played as .wav files, and then the four match choices appeared onscreen, with nothing in the center of the screen.

There were four conditions in this experiment. During an annual test each condition was started and completed before another condition began (see Fig. 1 for some examples). In the condition Photograph-to-Lexigram, a photograph was presented at the start of a trial as the sample, and four lexigram match options were presented. In the condition Lexigram-to-Photograph, a lexigram was presented at the start of a trial as the sample, and four photograph match options were presented. In the condition English-to-Lexigram, a spoken English word was presented at the start of a trial as the sample, and four lexigram match options were presented. In the condition English-to-Photograph, a spoken English word was presented at the start of a trial as the sample, and four photograph match options were presented.

In each condition, each chimpanzee completed 4 trials with each sample. For the years 1999, 2000, and 2001, there were 186 samples. For all subsequent years, there were 200 samples (Table 1). Within a test session, specific sample images or .wav files were randomly selected from this 186- or 200-item set. Incorrect match choices also came from the same 186- or 200-item set and were randomly chosen on each trial. The condition order for each chimpanzee was randomly determined each year. Only one condition was presented per session to each chimpanzee. The experimenter typically was seated behind the computer when testing a single chimpanzee or walked between two test areas when testing multiple chimpanzees. Critically, the experimenter could not see the computer screen on any trials, and thus was unaware of the sample image (although he or she could hear auditory samples), the match options, or the chimpanzees’ choices as they were being made, and thus could not inadvertently cue the chimpanzees during their responses. After hearing auditory feedback from the program, the experimenter typically (but not always) rewarded the chimpanzee on correct trials with fruit and other preferred and approved food rewards.

At the beginning of this experiment, Panzee had been the only one of the subjects to ever shown any proficiency with spoken English words [26, 30–31]. Thus, the other three chimpanzees were not tested in the two spoken English conditions because working in these conditions (often for a few weeks continuously, and at chance levels) produced frustration on their part, as indicated by less willingness to work. Mercury was tested mainly as a control participant to indicate how well a chimpanzee might perform in this study when basically choosing at random on each trial. He also showed no indication of performing above chance levels, and so was not tested every year to avoid frustrating him, especially during the early years of the project. In later years, he appeared excited to work on the task despite performing poorly, and so he was tested in those years. Thus, Lana, Sherman, and Panzee completed the testing in all 10 years (1999–2008) for the visual conditions, and Panzee participated for all 10 years for the auditory stimuli. Mercury completed testing in the two visual conditions in 2001 and from 2003–2008.

### Scoring

Proficiency with a given word was determined in the following manner. In each of the four conditions, a sample word was presented for four trials within the full test of that condition. For each word, proficiency with visual forms of the lexigram and its referent using the Photograph-to-Lexigram and Lexigram-to-Photograph conditions was assessed. For auditory proficiency (i.e., speech perception), performance in the English-to-Photograph and English-to-Lexigram conditions was examined.

Within each pair of conditions, if a chimpanzee was correct on fewer than three of four trials for either condition, that word was considered to be “Not Known” by the chimpanzee in that domain (visual or auditory) for that testing year. If the chimpanzee was correct on at least three of four trials in both conditions, the word was considered to be “Known” in that domain. These criteria were set because 6 of 8 trials correct (i.e., 3 of 4 trials in each condition) with a chance level of 25% exceeds chance performance (*p* < .05). However, 6 correct responses overall were considered to be inadequate when these responses were the result of being correct on 4 of 4 trials in one condition and 2 of 4 trials in the other because the latter case did not indicate high enough performance. This restriction provided a more conservative set of criteria. Thus, the chimpanzee had to show high performance in both conditions for a word to be included as part of that chimpanzee’s yearly vocabulary.

### Ethics Statement

This study was carried out in strict accordance with the recommendations in the Guide for the Care and Use of Laboratory Animals of the National Institutes of Health. The protocol for computerized testing of chimpanzees as used here and in all other studies that included these chimpanzees was reviewed and was approved by the Institutional Animal Care and Use Committee of Georgia State University (Protocol Number: A10021). The chimpanzees’ health and wellbeing was under continual monitoring by the Georgia State University veterinarian and by the animal care staff, who evaluated the chimpanzees frequently (multiple times each day). There was no discomfort experienced by the chimpanzees at any point in the study. They had continuous access to environmental enrichment including access to the outdoor yards in which there were climbing towers, ropes, hammocks, swings, and toys with which they could interact. There was no terminal endpoint to the study, and no use of euthanasia.

## Results

**Fig 2:**
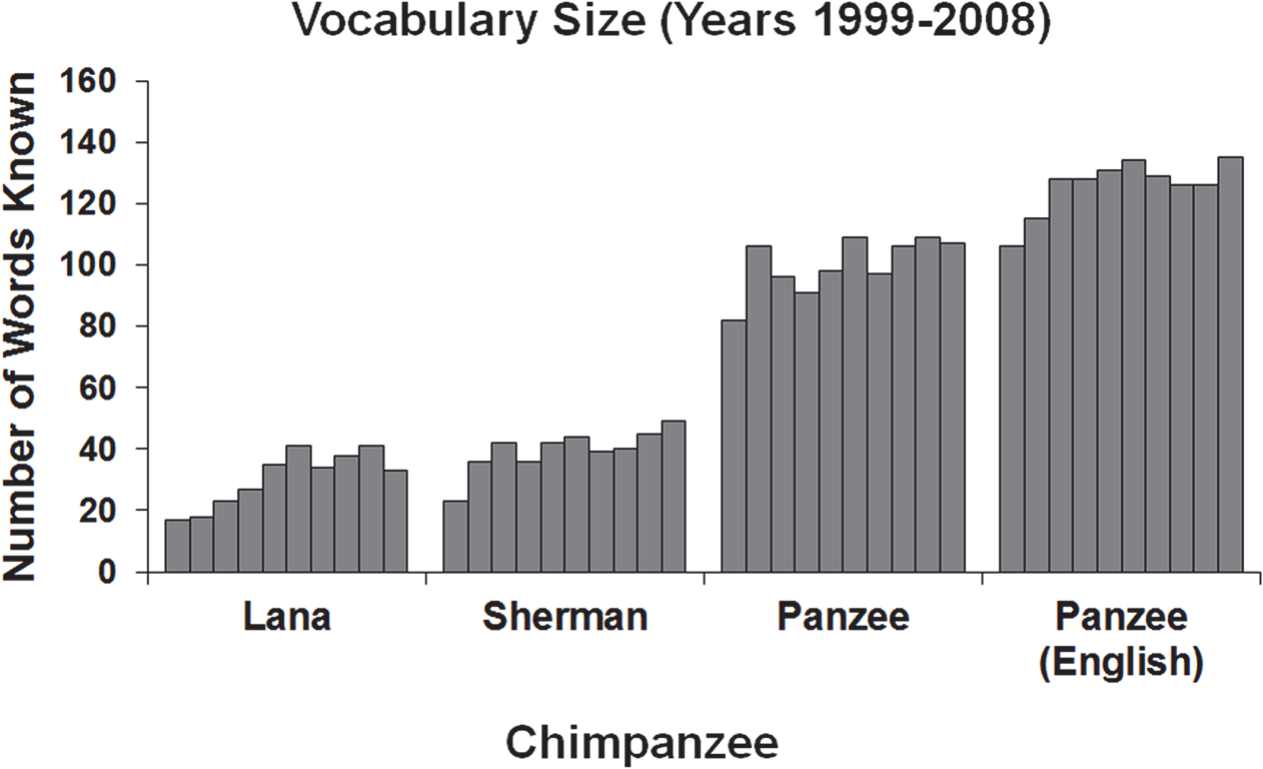
Number of Known words in each testing year for each chimpanzee. For Panzee, this is shown for visual samples and spoken English samples whereas for Lana and Sherman it is shown only for visual samples.

Fig. 2 presents the cumulative totals of Known words for each of the 10 years of testing for Lana, Sherman, and Panzee (and, for her, this includes visual and auditory sample stimuli). Mercury is not included in the figure because he never met criteria for any word in any year’s test, thereby confirming that the criteria were sufficiently conservative to not overestimate proficiency of word knowledge by chimpanzees that had not learned those words through past experiences.

Next, the correlations between performance in each year to each of the other years in terms of classifying each of the words as being Known or Not Known were examined. These correlations are shown in Table 2 for Lana and Sherman and in Table 3 for Panzee. For these three chimpanzees, every correlation within the matrix was statistically significant (all *p* < .01).

**Table 2:** Correlational matrices for Sherman and Lana comparing performance (Known or Not Known) between all pairs of years for all words that were tested.

Lana	2000	2001	2002	2003	2004	2005	2006	2007	2008
1999	.717	.674	.664	.563	.461	.478	.496	.478	.547
2000		.761	.794	.680	.616	.598	.520	.591	.670
2001			.819	.697	.549	.625	.590	.649	.694
2002				.739	.669	.600	.592	.561	.731
2003					.673	.627	.656	.664	.728
2004						.594	.670	.601	.608
2005							.629	.627	.731
2006								.638	.746
2007									.675
									
**Sherman**	2000	2001	2002	2003	2004	2005	2006	2007	2008
1999	.725	.696	.684	.656	.646	.631	.713	.636	.608
2000		.777	.793	.809	.797	.812	.778	.816	.811
2001			.842	.816	.832	.794	.794	.849	.781
2002				.777	.768	.734	.754	.850	.772
2003					.771	.794	.762	.849	.780
2004						.744	.760	.841	.848
2005							.732	.762	.776
2006								.748	.674
2007									.834

Note. All correlations were statistically significant, *p* < .01. The df for correlations that included years 1999–2003 was 184. The df for correlations that only included years 2004–2008 was 198.

**Table 3:** Correlational matrices for Panzee for visual and spoken English conditions comparing performance (Known or Not Known) between all pairs of years for all words that were tested.

Visual	2000	2001	2002	2003	2004	2005	2006	2007	2008
1999	.487	.510	.409	.473	.456	.405	.400	.447	.452
2000		.529	.420	.395	.484	.397	.452	.538	.420
2001			.453	.505	.474	.526	.528	.463	.582
2002				.432	.452	.319	.458	.472	.550
2003					.427	.332	.460	.308	.406
2004						.465	.447	.516	.477
2005							.453	.525	.423
2006								.508	.508
2007									.517
**Spoken**	2000	2001	2002	2003	2004	2005	2006	2007	2008
1999	.480	.560	.517	.484	.548	.553	.492	.480	.372
2000		.594	.640	.582	.571	.630	.594	.606	.472
2001			.624	.607	5.89	.660	.594	.606	.472
2002				.657	.626	.663	.677	.569	.547
2003					.541	.591	.662	.601	.541
2004						.657	.629	.541	.557
2005							.730	.600	.623
2006								.678	.618
2007									.596

Note. All correlations were statistically significant, *p* < .01. The df for correlations that included years 1999–2003 was 184. The df for correlations that only included years 2004–2008 was 198.

To establish a long-term vocabulary for each chimpanzee only those words that were known (based on meeting the yearly criteria) for at least 8 of 10 years were included. These words are shown in Table 4. A comparison of the yearly average number of words known and the number of words known for at least 8 of 10 years provides a type of “savings measure” of retention across time. Lana averaged 30.7 words known per year, and she had 17 words that were known for at least 8 of 10 years, a savings measure of 55%. Sherman averaged 39.6 words known per year, and he had 32 words that were known for at least 8 of 10 years, a savings measure of 80.8%. For visual samples, Panzee averaged 100.1 words known per year, and she had 68 words that were known for at least 8 of 10 years, a savings measure of 70%. For spoken English samples, Panzee averaged 125.8 words known per year, and she had 107 words that were known for at least 8 of 10 years, a savings measure of 85%. For Panzee, the number of words known was significantly higher over this 10-year period for the English samples than for the visual samples, paired-samples *t*(18) = 6.46, *p* <.001.

**Table 4:** The list of Known words in at least 8 of 10 testing years for each chimpanzee.

**Lana**					
Banana	Bread	Bug	Carrot	Chow	Coffee
Coke	Juice	Lighter	M&M’s	Milk	Orange
Perrier	Spoon	Straw	Strawberries	TV	
**Sherman**					
Apple	Banana	Blanket	Bread	Carrot	Cereal
Chow	Coffee	Coke	Collar	Fire	Ice
Juice	Keys	Lemonade	Level	Lighter	M&M’s
Magnet	Melon	Money	Orange	Orange drink	Pineapple
Raisin	Shot	String	Sweet potato	Tv	
**Panzee**	**Visual**				
A-frame	Apple	Apricot	Ball	Balloon	Banana
Blackberries	Blueberries	Book	Bowl	Bread	Bubbles
Bug	Camera	Carrot	Cheese	Cherries	Chow
Coke	Crisscross	Dog	Egg	Fire	Flat rock
Grapes	Hilltop	Hotdog	Ice	Jell-O	Jelly
Juice	Keys	Kiwi	Lemon	Lemonade	Light
Lookout	M&M’s	Melon	Midway	Milk	Mush Trail
Observation	Onion	Orange drink	Paint	Peaches	Peanut
Pear	Pineapple	Popsicle	Potato	Raisin	River
Rubber band	Sand pile	Shoe	Snake	Sparkler	Strawberries
Sugar	Sugarcane	Surprise	Taco	Toothpaste	Vacuum
Water	Yogurt				
**Panzee**	**Spoken**				
Apple	Apricot	Backpack	Ball	Balloon	Banana
Blackberries	Blueberries	Book	Bowl	Bread	Bubbles
Bug	Bunny	Butter	Carrot	Celery	Cereal
Chase	Cheese	Cherries	Chicken	Chow	Clay
Cover	Coffee	Coke	Colony room	Crisscross	Dog
Egg	Fire	Gorilla	Grapes	Green beans	Groom
Hat	Hide	Honeysuckle	Hotdog	Hurt	Ice
Jell-O	Jelly	Juice	Keys	Kiwi	Koolaid
Lemonade	Lettuce	Light	Lighter	Lookout	M&M’s
Matata	Melon	Milk	Mush Trail	Noodles	Observation
Oil	Onion	Orange	Orange drink	Orange juice	Paint
Peaches	Peanut	Pear	Phone	Pineapple	Pinecone
Pine needle	Plastic bag	Popsicle	Potato	Rain	Raisin
River	Rock	Rubber band	Shirt	Shoe	Shot
Snake	Soap	Sparkler	Spoon	Stick	Strawberries
String	Sugar	Sugarcane	Surprise	Sweet potato	Tickle
Tomato	Toothbrush	Toothpaste	Towel	Trash	Tv
Vacuum	Vitamins	Wash	Water	Yogurt	

Additionally, one might ask how the chimpanzees fared on their known words for the very first presentation of those words each year. If a chimpanzee missed Trial 1, the chimpanzee had to be correct on all remaining trials with that word, or it would not meet the criterion. However, this still left open the possibility that the chimpanzees relied heavily on feedback from that first trial to guide future responses to that sample each year. To assess this possibility, we examined performance on Trial 1 of each condition for each word that eventually was scored as being Known. We did this for the data from year 2000, although the same trend was seen in the other years as well. In 2000, Lana met criterion for 18 words, meaning there were 36 first presentations of a sample (18 when it was a photo sample, and 18 when it was a lexigram sample). For these 36 trials, Lana selected the correct match option on 34 trials (94.4% correct). Sherman met criterion for 36 words, generating 72 first presentations. Sherman selected the correct match option on 67 trials (93.1% correct). For visual samples, Panzee met criterion for 106 words, generating 212 first presentations. She selected the correct match option on 201 trials (94.8% correct). For auditory samples, Panzee met criterion for 115 words, generating 230 first presentations. She selected the correct match option on 216 trials (93.8% correct). Thus, all chimpanzees performed at very high levels on Trial 1 presentations of words that eventually were scored as being known for that year’s testing.

## Discussion

Across a decade of testing, these three chimpanzees showed a consistent degree to which their vocabulary sizes remained the same. The data indicate that all three chimpanzees retained the meanings of varying numbers of symbols that they had learned when younger and that, in some cases, are now symbols that have little or no use in their present lives (e.g., the location names for Panzee because she no longer visits those places).

The vocabularies of the three chimpanzees varied substantially. Lana clearly had the smallest vocabulary throughout this time range, and she also had the lowest savings measure of the three chimpanzees. She is only slightly older than Sherman (44 years to 41 years presently), and so it would not seem that age is the relevant factor. Rather, it is likely the case that Lana’s retention was not as strong because of the difference in how she was trained to use and respond to lexigrams compared to the other two chimpanzees. Lana was trained to produce sequences of responses, many of which were lexigrams that were part of the necessary grammatical structure of her computerized system. Thus, Lana had a smaller vocabulary for actual objects and foods even when she was actively involved early in life in her language acquisition project compared to Sherman and Panzee. This likely impacted her ability to retain as many lexigrams. In addition, some of Lana’s lexigrams from her original participation in the language acquisition project were removed from the lexigram keyboard in use at the LRC, and this occurred many decades ago. Therefore, she has not had the same exposure to seeing her original visual symbols that the other two chimpanzees have had through the present time. Importantly, Lana was given another long-term memory test for some of those original lexigrams, and she showed at that time that she had remembered many of those lexigrams for more than 20 years, even when those were not present on a daily basis [25]. Thus, Lana likely has a fairly good memory for some symbols, but probably was limited in how large her long-term vocabulary could be given her more limited original vocabulary.

Sherman showed a greater degree of retention, and he (and Panzee) also showed an impressive degree of consistency in known words across years. Sherman’s more “flexible” use of lexigrams, with the emphasis of productive use and comprehensive use of lexigrams during his early years in the project [18] may have contributed to his good memory for many of these lexigrams. Importantly, Sherman showed retention of some items that are rarely, if ever, present between the test intervals. Lever, Magnet, Money, and String were all objects that were important parts of daily routines when he was younger, but those are items that are not presently used with him, and they have not been used in his daily keyboard interactions with caretakers for more than 20 years, even though those lexigrams remain on the keyboard that he uses. However, most of Sherman’s present vocabulary consists of food items, and these are things that he can request on a daily basis. This indicates that for Sherman retention is likely affected by salience of the word and the need to use the symbols frequently, a point that is also true for Lana.

Panzee’s vocabulary, both in the visual domain and for spoken English words, was more impressive. This is not just because of its size, but also for its content. Although Panzee retained the meaning for a large number of food lexigrams, she also retained the meaning of many objects, some of which were rarely if ever experienced in her daily routines over the past 20 years (e.g., Ball, Balloon, Bubbles, Fire, Rubber band, Sparkler). She also retained the lexigram names for many locations, and these are places that Panzee has not seen in 20 years as well (A-frame, Crisscross, Flat rock, Hilltop, Lookout, Midway, River). These were highly salient locations when she was younger and was able to travel daily to those places, often through initiating group movements by requesting through lexigram choices that everyone go to those locations.

For Panzee, speech comprehension has remained particularly strong throughout this decade of testing. This is an important finding because few of the symbol-trained apes showed strong speech comprehension, with the best examples being the bonobos that also were trained with the lexigram system [30–31, 33–34, 43–44]. Panzee’s results show that early acquisition of speech comprehension lasts a lifetime, and in fact her speech comprehension outpaced her lexigram retention. This might have been the result of how differently speech comprehension and lexigram comprehension were valued by Panzee relative to Sherman and Lana. For Lana, speech comprehension was not relevant given the nature of the computerized apparatus to which she had to respond. It did not produce speech, and so there was little or no opportunity for Lana to associate spoken words with real objects or lexigrams. Sherman (and Austin) were exposed to more human speech, but it was not specifically a focus of their acquisition projects, and so often they would work in silence on various tasks. However, Panzee (like many of the bonobos given the same rearing) was immersed in speech from the beginning of her symbol training. Lexigram use by the people around her was accompanied by speech, providing her with multiple perceptual routes for processing symbolic indications of real world events and objects. One critical aspect of this type of rearing is that Panzee, unlike Lana and Sherman, did not have to attend to all visual lexigram stimuli that were presented to her, as she also had the auditory information that she could process. She often did just that, ignoring lexigram use by humans if she instead could attend to the speech that she heard. This might have led to Panzee prioritizing speech stimuli over visual stimuli during communicative interactions, although there are certainly plausible alternate explanations for why her speech comprehension has outpaced her lexigram retention.

Although he showed no capacity for performing this task in any of the years in which he was tested, Mercury served a critical role in this longitudinal study. Mercury is highly trained to perform computerized tasks, and in areas outside of symbol use typically performs the same as the other chimpanzees in basic tests of learning and cognition [45–50]. Thus, if the use of reward for correct responses would “stamp in” new learned associations between stimuli in this task, Mercury would have shown this outcome. That he did not instead suggests that the other chimpanzees were not learning new associations, but instead were demonstrating their memory for associations between lexigrams and their referents that had been acquired many years before this testing began. Mercury thus serves to show that this testing routine, conducted only once per year, and then for only 4 trials per stimulus in each condition, did not produce good performance unless a chimpanzee already knew and remembered the association between these lexigrams and their referents.

An important point to note is that, as shown in Fig. 2, there was an increase in the number of items that Lana, Sherman, and Panzee (for English words) met criterion with in the early years of this study. This might suggest some effect of learning, as the chimpanzees were rewarded for correct responses during these tests. Alternately, the chimpanzees may simply have remembered more lexigrams with each year’s testing. We cannot distinguish between these two possibilities. However, as Mercury showed no such improvements over time this makes it seem unlikely that truly new associations were formed as a result of the testing. Instead, we suggest that perhaps the chimpanzees’ memory for some lexigrams was not very strong, but got stronger with the feedback that the chimpanzees received when responding to these stimuli in the early years. Thus, both memory and learning likely played a role in performance in the early years of the project. The results indicate that these chimpanzees did remember their symbols, but with varying success, and there was evidence that memory improved over time perhaps due to the feedback that the chimpanzees received while testing.

Documenting the continued lexigram and speech comprehension of these chimpanzees is more than an academic exercise. Symbol comprehension typically precedes and in some cases exceeds the symbol production of nonhuman animals and young human children [34, 43], and the retained abilities of our chimpanzees offer insights into the robustness of comprehension over time. Although none of the chimpanzees is part of active “language acquisition” research any longer, their vocabularies have proved to make them a truly unique scientific resource. In some ways, they are equivalent to those few human participants with such rare qualities that they can become the center of entire research programs (e.g., patients such as H.M. and others [51–52] and “split brain” individuals who have undergo corpus calloscotomy [53]). Researchers make use of the abilities of these chimpanzees to communicate symbolically to assess other areas of cognitive ability. For example, apes that use lexigrams can communicate about past events that they experienced or about future intended actions [54–55]. The use of lexigrams as relevant task stimuli allow for asking questions about the role of symbolic rewards in areas such as self-control and delay of gratification [56–57], prospective memory [58–59], metacognition [60], bartering and exchange behavior [61], and analogical reasoning [62]. In these areas, the symbol-trained chimpanzees have shown positive results of using symbols to facilitate self-control, to show prospective memory and metacognition, and they engage in limited forms of analogical reasoning and bartering. Also, lexigram symbols allow one to ask questions with chimpanzees that can be directly related to areas of human cognition research that rely heavily on language and symbolic stimuli, such as the Stroop effect, which Lana also shows [63], and learning through exclusion (or what is sometimes called fast mapping, an ability that did not emerge in these chimpanzees as they showed exclusion, but no long-term learning through use of exclusion [41]. Additionally, the speech comprehension shown by Panzee has allowed for truly unique assessments of animal cognition and perception such as her cross modal use of exclusion [38], and her perception of degraded speech [32].

There are only a few living apes that can provide these kinds of insights into cognition, and the evolution of some of the hallmark cognitive processes that underlie the mental abilities of modern humans. Unfortunately, it does not appear that this kind of intensive research that involves years of commitment to produce such symbolic competencies will continue in the future, and many of the still-living apes that once were in such symbol-acquisition studies are no longer active in mainstream research. There are ongoing assessments of the capacities of some of the chimpanzees that were trained in sign-language, including assessments of their comprehension and use of signs, as well as other aspects of communication by those apes [64–68], but many of those apes also have died. Thus, these few remaining lexigram-trained chimpanzees continue to offer great benefit to psychological science, and hopefully will continue to actively engage in research and provide new insights into the role of symbol knowledge in other areas of cognition.

## Supporting Information

S1 Fig The ARRIVE Guidelines Checklist for Animal Research. This reports the pages on which each element of this guideline is addressed in reporting in vivo experiments.
(PDF)
